# Optimal molecular binding data and pharmacokinetic profiles of novel potential triple-action inhibitors of chymase, spleen tyrosine kinase, and prostaglandin D2 receptor in the treatment of asthma

**DOI:** 10.1186/s43141-023-00577-8

**Published:** 2023-11-10

**Authors:** Precious Ayorinde Akinnusi, Samuel Olawale Olubode, Ayomide Oluwadarasimi Adebesin, Adebowale Abiodun Alade, Victor Chinedu Nwoke, Sidiqat Adamson Shodehinde

**Affiliations:** 1 Department of Biochemistry, Adekunle Ajasin University, Akungba Akoko, Ondo Nigeria; 2 Department of Biochemistry, Cancer and Genomics Lab, Covenant University, Ota, Nigeria; 3 Department of Biochemistry, Enugu State University of Science and Technology, Enugu, Nigeria

**Keywords:** Asthma, Spleen tyrosine kinase, Chymase, Prostaglandin D2 receptor, Molecular docking, Pharmacokinetics, Drug discovery

## Abstract

**Background:**

Asthma is a chronic and complex pulmonary condition that affects the airways. A total of 250,000 asthma-related deaths are recorded annually and several proteins including chymase, spleen tyrosine kinase, and prostaglandin D2 receptor have been implicated in the pathophysiology of asthma. Different anti-inflammatory drugs have been developed for the treatment of asthma, particularly corticosteroids, but the associated adverse reactions cannot be overlooked. It is therefore of interest to identify and develop small molecule inhibitors of the integral proteins associated with asthma that have very little or no side effects. Herein, a molecular modeling approach was employed to screen the bioactive compounds in *Chromolaena odorata* and identify compounds with high binding affinity to the protein targets.

**Results:**

Five compounds were identified after rigorous and precise molecular screening namely (−)-epicatechin, chlorogenic acid, ombuine, quercetagetin, and quercetin 3-O-rutinoside. These compounds generally showed impressive binding to all the targets understudy. However, chlorogenic acid, quercetagetin, and quercetin 3-O-rutinoside showed better prospects in terms of triple-action inhibition. Further pulmonary and oral pharmacokinetics showed positive results for all the reported compounds. The generated pharmacophore model showed hydrogen bond donor, hydrogen bond acceptor, and aromatic rings as basic structural features required for triple action inhibition.

**Conclusion:**

These findings suggest that these compounds could be explored as triple-action inhibitors of the protein targets. They are, therefore, recommended for further analysis.

## Background

Asthma is an acute and complex airway disease described by hyperresponsive airways (AHR) and eosinophilic inflammation. Lung biopsy examination in bronchial asthma reported the presence of mast cells, eosinophils pulmonary infiltration, lymphocytes, and macrophages. Furthermore, morphological alteration in asthmatic bronchi’s extracellular and cellular parts, including epithelial hypertrophy, subepithelial fibrosis, cell hyperplasia, thickening of the airway wall, and myofibroblast hyperplasia, are all termed airway remodeling [1, 2]. It is a global disease commonly found in developing countries with an increasing incidence. A total of 250,000 people have been associated with asthma-related deaths annually. There is a tendency that 400 million people will be affected globally by 2025 [3, 4]. The use of corticosteroids, an anti-inflammatory drug to treat asthma, has posed various adverse effects on the health of individuals, including growth retardation in younger ones, inhibited hypothalamic-pituitary axis, and high infection threat [5, 6].

Spleen tyrosine kinase (Syk) is a non-receptor protein tyrosine kinase found in the cytoplasm that triggers varieties of respiratory inflammatory responses by activating (immunoglobulin E) IgE [7], releasing eosinophil mediators, and producing eicosanoid and cytokine [8, 9]. Syk is activated when the SH2 tandem domains attached to FcεRIITAMs (immunoreceptor tyrosine-based activation motifs) phosphorylate and transform FcεRI signaling in mast cells [10]. Activated Syk modulates numerous signal transduction molecules upstream and triggers asthma immune responses, such as protein kinase C (PKC), cytosolic phospholipase A2 (cPLA2), and nuclear factor kappa-B (NF-kB) [11]. As a result, Syk promotes immune-mediated diseases such as asthma and allergies. Inhibition of Syk activation change mast cell degranulation and leukocyte immune response [12, 13] and reduce inflammation in vivo. However, because Syk is positioned upstream in the signaling cell cascade of several immunological receptors, therapeutics that target Syk may be more effective. Hence, targeting Syk is a crucial target for asthma treatment.

Prostaglandin D2 (PGD2) is a key cyclooxygenase product generated mostly by mast cells; higher levels of PGD2 have been seen in asthmatics [14, 15]. PGD2 effects were related to the activation of the prostaglandin D2 receptor (PD2), also termed CRTH2 (chemoattractant receptor-homologous molecule expressed on Th2 cells), and a high level of thromboxane (TP) receptor activities [16]. PGD2 signaling activates cytokine generation and chemokinesis migration in leukocytes, including T cells, basophils, eosinophils, and type 2 innate lymphoid cells (ILC2s). The PGD2–CRTH2 pathway enhances in vitro ILC2 migration and build-up in the lung in vivo, thus leading to the development of type 2 lung infection. ILC2 levels are elevated in asthmatic airways and induce eosinophilia through type 2 cytokine production [17]. A potential therapeutic target for different acute inflammatory airway disorders treatment related to ILC2 responses, such as allergic rhinitis, chronic obstructive pulmonary disease, and asthma, is the PGD2–CRTH2 pathway [18–22].

Chymase is a monomeric protease that cleaves various substrates such as fibronectin, pro-MMP-2, MMPs, pro-MMP9, IL-13, IL-15, and IL-33 [23, 24]. When mast cells (MC), which play an essential role in inflammation conditions including asthma, are activated, several inflammatory compounds such as growth factors, cytokines, histamine, and a large number of different MC-restricted proteases, like carboxypeptidase A3 (CPA3), chymase, and tryptase, are released into the extracellular space [25–27]. Chymase cleaves to different substrates and changes the modification of the extracellular matrix compounds, such as chymase degrades fibronectin released from Human lung fibroblast (HLF) [23]. It induces pro-MMP-2, produced by the fibroblast airway [28, 29]. Thus, chymase is involved in extracellular matrix remodeling [30] and also causes an effect on primary human airway fibroblasts and their morphologic features, thereby contributing to inflammation [31]. It is crucial to develop strategies to inhibit chymase activity.

*Chromolaena odorata*, a perennial plant of the family Asteraceae, is widely prevalent in many parts of the world. It is otherwise known as Siam weed [32]. Studies showed that the plant contains flavonoids, glycosides, and saponins, which allow it to demonstrate a comprehensive series of pharmaceutical actions such as antidiabetic, anti-inflammatory, astringent, antimalarial, and antifungal properties [33–35] by interfering with carbohydrate metabolism. The biological actions of flavonoids in the treatment of diseases have piqued the interest of researchers. *C. odorata* is a potent anti-inflammatory agent because of its ability to hinder the inflammatory pathway, thus preventing chronic inflammation and inhibiting prostaglandin-mediated inflammation [36, 37]. To this end, we propose that the compounds of *C. odorota* may be active in the treatment of asthma through their inhibitory interactions with chymase, spleen tyrosine kinase, and prostaglansin 2D receptor. As a result, the present research employed biomolecular simulation methods, including molecular docking, molecular mechanics generalized born surface area, absorption, distribution, metabolism, excretion studies, and pharmacophore modeling, to predict the inhibitory activities of *C. odorata* bioactive constituents in the management of asthma.

## Materials and methods

### Ligands and protein targets

To identify potential triple-action inhibitors of chymase, spleen tyrosine kinase, and prostaglandin D2 receptor, the compounds of *Chromolaena odorata* were mined from PubChem in 2D sdf format [38]. Subsequently, the crystal structures of the chymase (PDB ID: 3SON), spleen tyrosine kinase (PDB ID: 4PV0), and prostaglandin D2 receptor (PDB ID: 6D26) were downloaded from the protein data bank (http://www.rscb.org/) [39–41].

### Ligand preparation

The bioactive compounds of *Chromolaena odorata* to be used in molecular docking were initially prepared using the LigPrep tool. This was done by generating their ionization states and tautomers at pH = 7.2 ± 0.2. and optimization employing the OPLS 2005 force field (Schrodinger release 2017).

### Protein preparation and receptor grid generation

The protein structures were incorporated in Maestro and subsequently prepared using the protein preparation wizard. Missing side chains were added using prime, waters, and other bound moieties (non-standard ligands) were deleted, hydrogen positions were optimized, and restrained energy minimization was performed on the proteins. Furthermore, to guide the automated docking procedure, grid boxes were generated with respect to the position of the co-crystallized ligand of the proteins.

### Molecular docking

The molecular docking procedure was carried out using the Glide script on Maestro 11.1 [42]. The compounds were docked into the previously prepared grid of the protein targets to identify compounds with potent inhibitory interactions with the proteins [43]. The results from the most rigorous screening (XP) were exported for further analysis. Also, the docked ligand-receptor complexes were exported and the amino acid interactions were visualized using the BIOVIA discovery studio visualizer [44].

In addition to our primary molecular docking investigations for the specified targets, we conducted a comparative study by including the co-crystalized ligands pertinent to each target, as well as three widely recognized standard inhibitors: chymostatin for chymase, cerdulatinib hydrochloride for spleen tyrosine kinase, and BI 671800 for the prostaglandin D2 receptor [45–47]. This extended analysis aimed to comprehensively assess and contrast the binding affinities and interactions of these compounds within the framework of our docking procedures.

### Pharmacophore modeling

PHASE graphical user interface in Schrodinger’s suite was employed to generate information on the molecular orientation of vital functional groups that are predominantly involved in the characteristic binding of the top-scoring ligands to the protein target [48].

### Pharmacokinetic profiling

The absorption, distribution, metabolism, excretion, and toxicity (ADMET) as well as the drug-likeness of the top-scoring compounds were analyzed using SwissAdme, Pro-Tox II, and Admetlab 2.0 online tools [49–51]. In addition to oral drug-likeness, pulmonary pharmacokinetic profiles were also studied. The pharmacokinetic descriptors analyzed include lipophilicity, water solubility, molecular weight, topological surface area, gastrointestinal absorption, pulmonary dissolution, permeability, volume distribution, blood-brain barrier permeation, P-glycoprotein substrate candidacy, inhibition of cytochrome P450 enzymes, plasma protein binding, carcinogenicity, LD50, hepatotoxicity, and clearance.

## Results

**Fig. 1:**
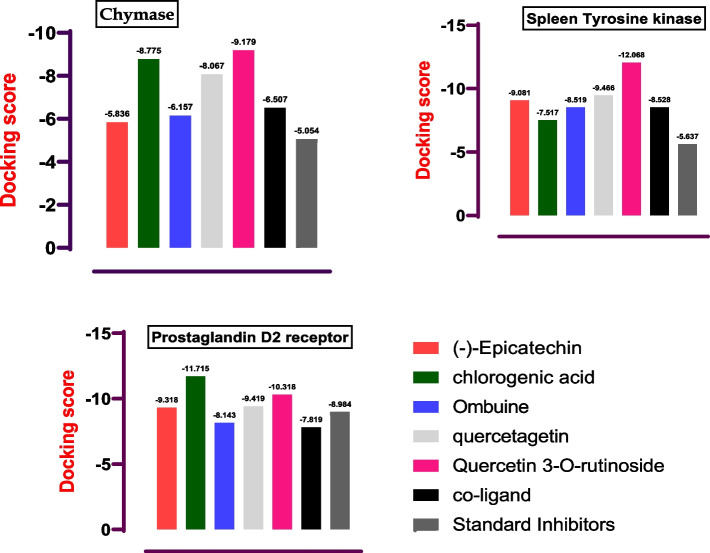
The docking scores of the lead compounds

The result of the molecular docking procedure for chymase (Fig. 1) showed a robust binding affinity for quercetin 3-O-rutinoside (− 9.179 kcal/mol), quercetagetin (− 8.067 kcal/mol), and chlorogenic acid (− 8.755 kcal/mol). Ombuine and (−)-epicatechin showed moderate binding affinities with docking scores of − 6.157 kcal/mol and − 5.836 kcal/mol, respectively. Chlorogenic acid, quercetagetin, and quercetin 3-O-rutinoside exhibited notably superior docking scores in comparison to the co-crystalized ligands. Remarkably, all the lead compounds demonstrated superior docking scores when evaluated against the standard compounds.

Similarly, quercetin 3-O-rutinoside had the highest binding affinity to spleen tyrosine kinase with a docking score of − 12.068 kcal/mol. (−)-Epicatechin, chlorogenic acid, ombuine, and quercetagetin also scored highly with docking scores of − 9.081 kcal/mol, − 7.517 kcal/mol, − 8.519 kcal/mol, and − 9.466 kcal/mol, respectively. Overall, all the reported compounds showed better prospects than the co-crystallized ligand and the standard compound.

All the reported compounds ranked higher than the standard co-crystallized ligand of the prostaglandin D2 receptor. Chlorogenic acid exhibited the highest binding affinity with a docking score of − 11.715 kcal/mol and quercetin 3-O-rutinoside also showed an impressive binding with a docking score of − 10.318 kcal/mol. (−)-Epicatechin, ombuine, and quercetagetin had docking scores of − 9.318 kcal/mol, − 8.143 kcal/mol, and − 9.419 kcal/mol, respectively, which are also considered good binding affinity to prostaglandin D2 receptor. However, the standard compound had a slightly higher docking score than ombulne.

**Fig. 2:**
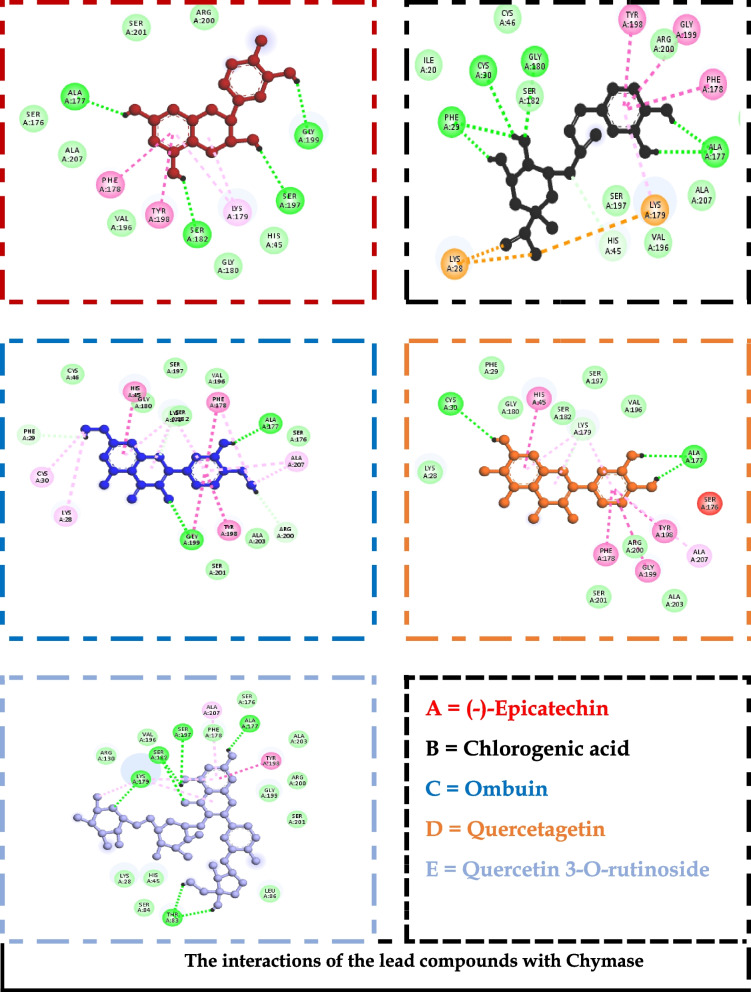
The interactions of the lead compounds with chymase

**Fig. 3:**
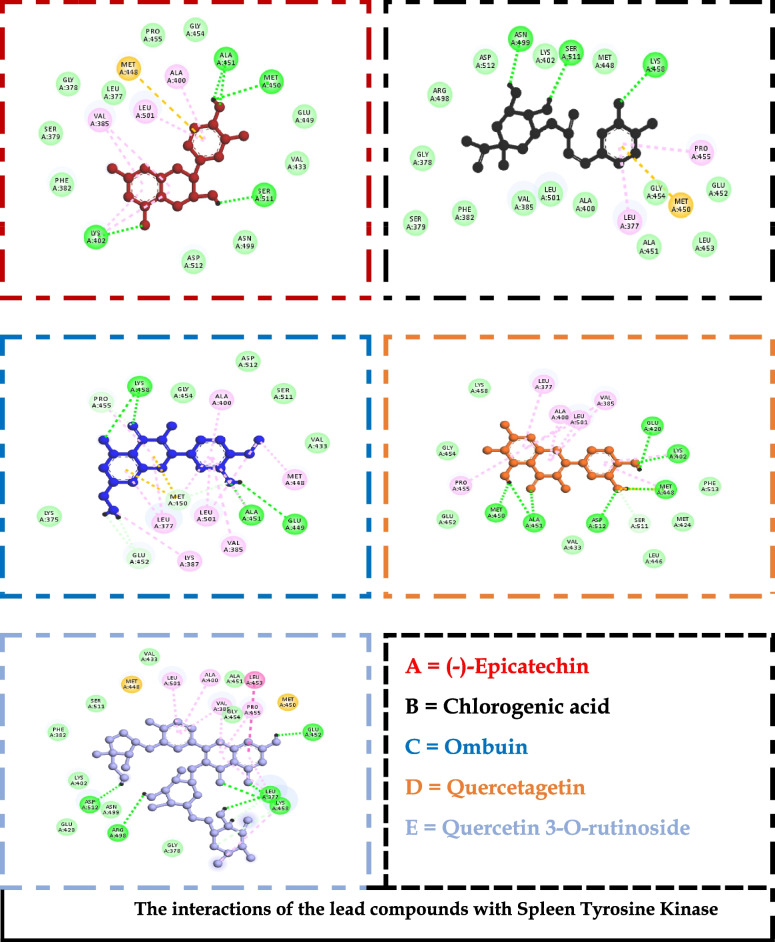
The interactions of the lead compounds with spleen tyrosine kinase

**Fig. 4:**
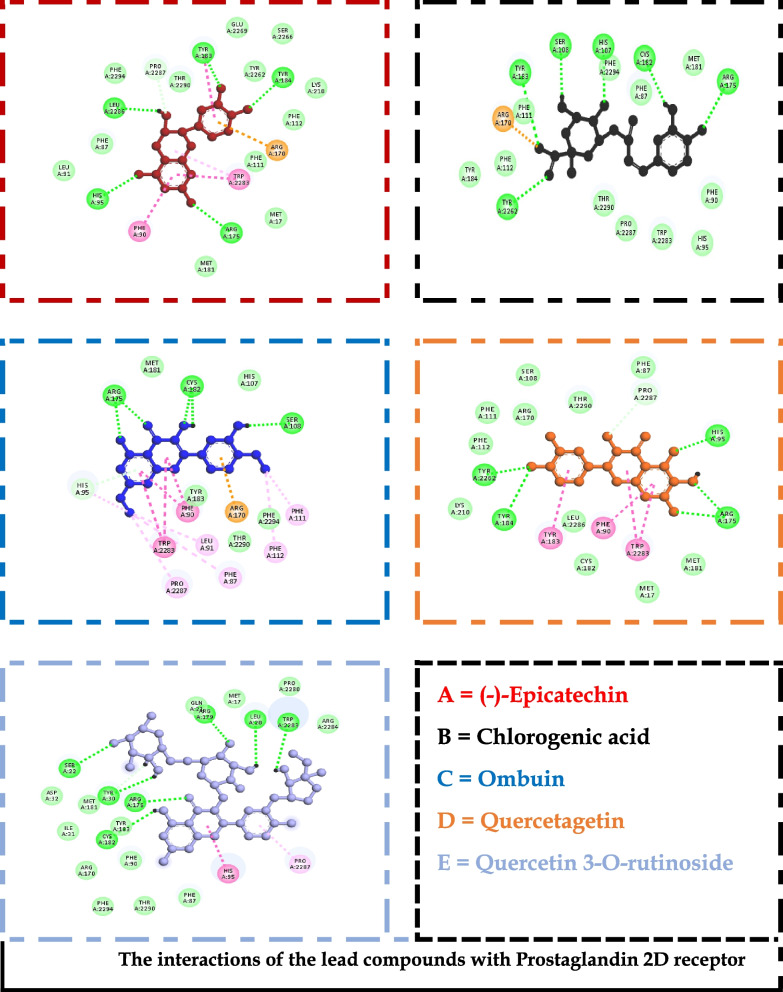
The interactions of the lead compounds with Prostaglandin 2D receptor

The generated receptor-ligand poses of the docked complexes were studied to reveal the specific interactions. Quercetin 3-O-rutinoside, the compound with the highest docking score against chymase, had interactions with THR^83^, LYS^179^, LYS^28^, SER^182^, SER^197^, ALA ^177^, and TYR^198^ (Fig. 2). Similarly, against Syk, specific contacts were made with GLU ^452^, LEU^453^, LEU^377^, LYS^458^, ARG^498^, ASN^499^, ASP^512^, and MET^338^ (Fig. 3). Also, chlorogenic acid had interactions with PHE^111^, ARG^170^, SER^108^, HIS^107^, CYS^182^, and ARG^175^ when complexed with the ligand-binding site of the prostaglandin D2 receptor (Fig. 4).

The physicochemical properties of the lead compounds are presented in Table 1. The log *P* value, as predicted by Admetlab 2.0, which represents the lipophilicity of the compounds ranged from − 0.763 to 1.588 with quercetin 3-O-rutinoside being the least lipophilic. The log *S* value which is a measure of water solubility ranged from − 3.928 to − 1.198. Reported also are the molecular weight of the compounds, topological surface area, and the number of hydrogen bonds.

**Table 1:** Physicochemical properties of the top-scoring compounds

Compounds	Molecular weight	TPSA	log *P*	log *S*	nHA	nHD
(−)-Epicatechin	290.08	110.38	1.142	−2.99	6	5
Chlorogenic acid	354.1	164.75	−0.162	−1.198	9	6
Ombuin	330.07	109.36	3.194	−3.78	7	3
Quercetagetin	318.04	151.59	1.588	−3.536	8	6
Quercetin 3-O-rutinoside	610.15	269.43	−0.763	−3.928	16	10

The absorption and distribution descriptors are reported in Table 2. The result showed high gastrointestinal absorption for (−)-epicatechin and ombuin. Also, (−)-epicatechin and quercetin 3-O-rutinoside were shown to be substrates of permeability glycoprotein. Notably, none of the compounds can permeate the blood-brain barrier. The volume distribution ranged from 0.351 to 0.754 with chlorogenic having the least distribution and quercetin 3-O-rutinoside having the highest distribution.

**Table 2:** Absorption and distribution descriptors

Compounds	GI absorption	HIA	P-gp substrate	ppB	VD (L/kg)	BBB penetration
(−)-Epicatechin	High	HIA+	Yes	92.06%	0.661	No
Chlorogenic acid	Low	HIA−	No	67.18%	0.351	No
Ombuin	High	HIA+	No	94.05%	0.718	No
Quercetagetin	Low	HIA+	No	95.28%	0.603	No
Quercetin 3-O-rutinoside	Low	HIA−	Yes	83.81%	0.754	No

According to swissAdme predictions (Table 3), chlorogenic acid, (−)-epicatechin, and quercetin 3-O-rutinoside are non-inhibitors of the essential CYP isoforms.

**Table 3:** Pharmacokinetic descriptors (CYP inhibition)

Compounds	CYP1A2 inhibitor	CYP2C19 inhibitor	CYP2C9 inhibitor	CYP2D6 inhibitor	CYP3A4 inhibitor
(−)-Epicatechin	No	No	No	No	No
Chlorogenic acid	No	No	No	No	No
Ombuin	Yes	No	Yes	Yes	Yes
Quercetagetin	Yes	No	No	No	Yes
Quercetin 3-O-rutinoside	No	No	No	No	No

The toxicity predictions (Table 4) revealed no toxic action in the respiratory system and in the liver. Also, the clearance rate ranged from 1.349 to 17.911 with quercetin 3-O-rutinoside having the least clearance rate and (−)-epicatechin having the highest.

**Table 4:** Toxicity and excretion

Compounds	CL	T_1/2_	LD50 (mg/kg)	Hepatotoxicity	Carcinogenicity	Respiratory toxicity
(−)-Epicatechin	17.911	0.853	10000	No	No	No
Chlorogenic acid	3.251	0.928	5000	No	No	No
Ombuin	4.929	0.843	5000	No	No	No
Quercetagetin	7.705	0.936	159	No	Yes	No
Quercetin 3-O-rutinoside	1.349	0.524	5000	No	No	No

The oral drug-likeness predictions of the lead compounds are shown in Table 5. (−)-Epicatechin, ombuin, and quercetegetin had a bioavailability score of 0.55. Chlorogenic acid returned a value of 0.11, and quercetin 3-O-rutinoside showed a value of 0.17

**Table 5:** Druglikeness and bioavailability score

Compounds	Lipinski rule	Pfizer rule	GSK rule	Verber rule	Bioavailability score
(−)-Epicatechin	+	+	+	+	0.55
Chlorogenic acid	+	+	+	−	0.11
Ombuin	+	+	+	+	0.55
Quercetagetin	+	+	+	−	0.55
Quercetin 3-O-rutinoside	−	+	−	−	0.17

Compounds with (+) obey the corresponding rule while those with (−) do not

## Discussion

In search of small molecule triple-action inhibitors of chymase, spleen tyrosine kinase, and prostaglandin D2 receptor CRTH2, the compounds of *Chromolaena odorata* were analyzed and docked against the ligand-binding sites of protein targets to reveal the individual binding affinities to the targets. The result of the molecular docking procedure revealed different compounds with potential inhibitory action on these targets. The compounds were analyzed and five lead compounds with the most robust triple-action activity were selected and reported herein. Spleen tyrosine kinase (Syk) has been shown to play essential roles in inflammatory responses in allergic asthma, and Syk inhibitors have previously evolved as part of a new anti-inflammatory strategy in treating asthma [3, 7, 52]. Therapeutic interventions that inhibit Syk may be more effective than treatments that focus on downstream targets [53]. Also, research has considered prostaglandin 2D receptor 2 as a predominant player in the propagation of asthma due to its role in initiating and amplifying the inflammatory response associated with the disease state. The receptor can be activated by both allergic and nonallergic agents, and several studies are of the opinion that antagonizing it is a good therapeutic intervention for asthma [54].

With respect to the intended triple activity, quercetin 3-O-rutinoside, quercetagetin, and chlorogenic acid are considered to be the most suitable inhibitors as per the generated heat map (Fig. 2). Quercetin 3-O-rutinoside showed the most robust triple affinity to the protein targets. (−)-Epicatechin and ombuine had impressive affinities for spleen tyrosine kinase and prostaglandin D2 receptor but moderate binding affinities for chymase. This makes the compounds less suitable than quercetin 3-O-rutinoside, quercetagetin, and chlorogenic acid. However, they can be explored as double-action inhibitors of both spleen tyrosine kinase and prostaglandin D2 receptor.

**Fig. 5:**
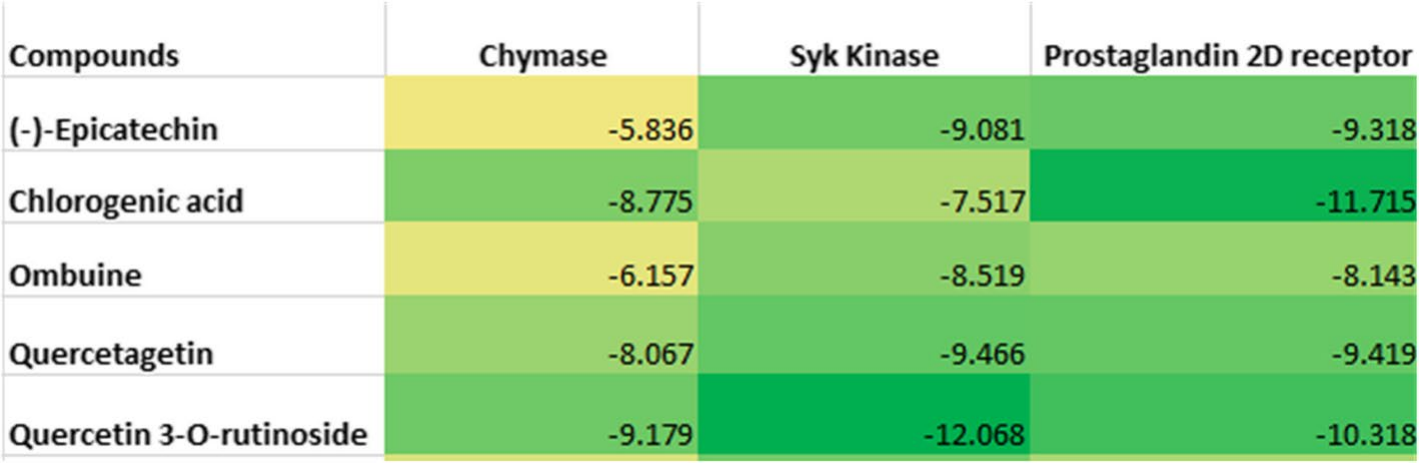
Heat map of the docking scores of top-scoring compounds

**Fig. 6:**
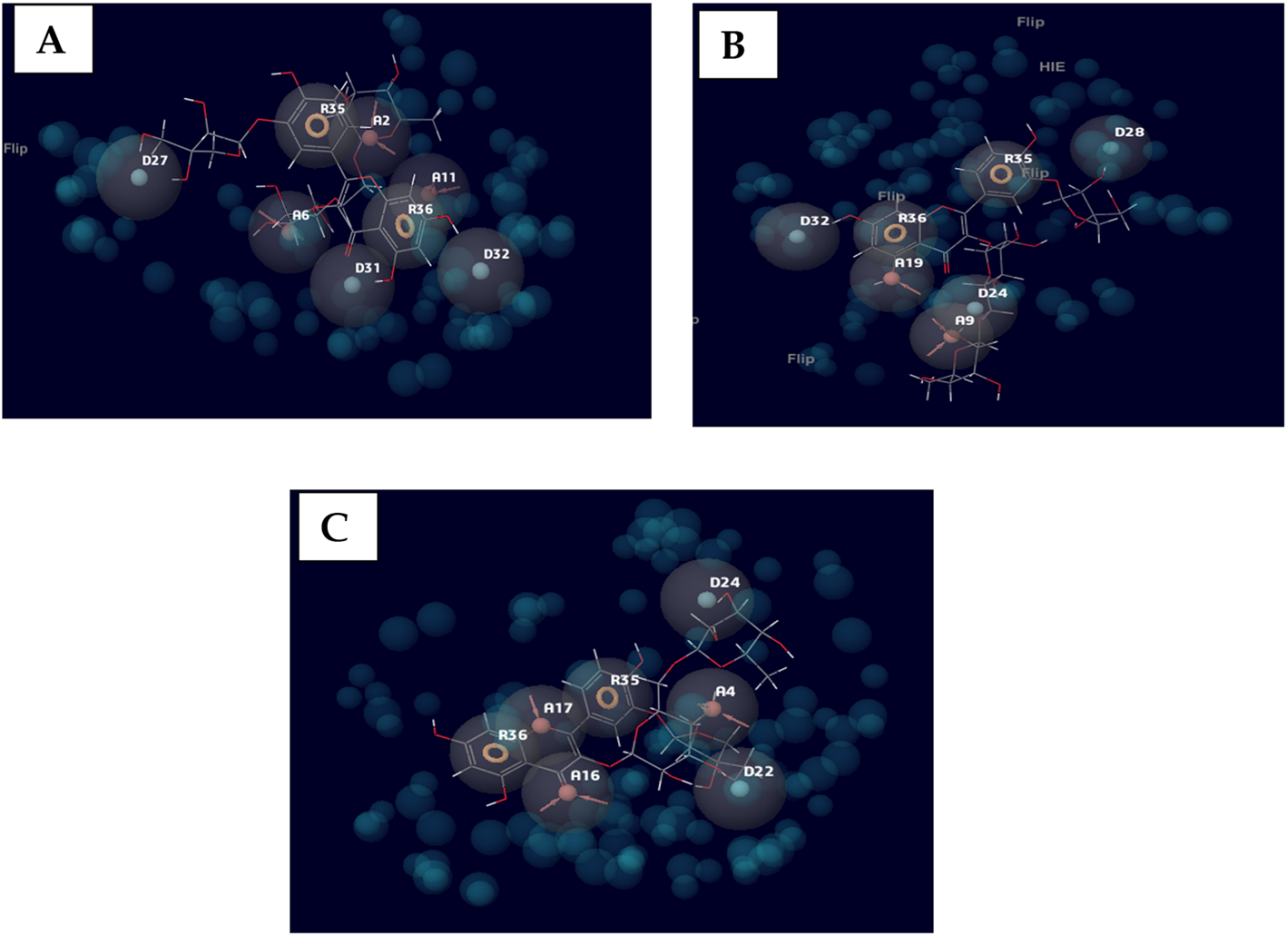
Pharmacophore models

To identify optimizable ligand-protein interactions, the amino acid interactions of the lead compounds with the binding pockets of all the protein targets were analyzed. The specific interactions in these complexes are shown in Figs. 2, 3, and 4 for chymase, spleen tyrosine kinase, and prostaglandin D2 receptor, respectively. Hydrogen bond is the most prevalent interaction observed, and this could be linked to the consideration that it is one of the most stabilizing and specific interactions in biological systems [55]. In addition to its role in the recognition of ligand-protein binding, hydrogen bond is also involved in the affinity of ligands for protein [56]. π-stacking interactions were also observed in some of the interactions. The interaction has been shown to contribute immensely to ligand binding. It also plays a vital role in medicinal chemistry because it can be seen between aromatic rings; an automatic ring from the ligand and another from aromatic amino acid residues in the binding pocket of proteins [55, 57] Figs 5 and 6.

## Pharmacophore model

Receptor-based pharmacophore hypothesis was developed from the complex formed by the binding of quercetin 3-O-rutinoside to the ligand-binding site of the targets. The specific details of the molecular arrangement of contributing functional groups that are involved in the specific recognition and characteristic binding of high-affinity compounds for the target can be obtained from the PHASE interface in Schrodinger’s suite [48].

The developed E-pharmacophore hypothesis from the binding of quercetin 3-O-rutinoside to the ligand-binding sites of the protein targets includes aromatic rings, H-bond acceptor, and H-bond donor interactions. Interestingly, this hypothesis is employable in forming the basic structural architecture that is common to all potential triple inhibitors of chymase, spleen tyrosine kinase, and prostaglandin D2 receptor CRTH2. Similarly, a fundamental skeletal structure of compounds with precise angles and distance that will bind strongly to the binding pockets of the proteins.

## Pharmacokinetic profiling

Pulmonary administration has in recent times been the subject of extensive exploration as a route for the treatment of local lung diseases such as asthma [58]. However, long pulmonary exposure is critical to achieving the desired therapeutic effect. Lipophilicity and solubility of compounds are the major contributors to the fast absorption and low pulmonary exposure of drugs. After being inhaled, hydrophilic drugs may rapidly dissolve in the fluid lining the lungs, diffuse through the lung epithelium, and move swiftly away from the lungs into systematic circulation through absorption [59]. Similarly, lipophilic drugs passively diffuse through the cells and induce faster absorption through the respiratory epithelium [60].

In this regard, quercetin 3-O-rutinoside is expected to have the highest pulmonary exposure due to its combination of lowest solubility (− 0.3928 log mol/L) and lowest lipophilicity (−0.763 log mol/L) (Table 1). Ombuine and quercetagetin also showed low solubility index with log *S* values − 3.78 log moL and − 3.536 log mol/L, respectively, and would have a longer dissolution time than (−)-epicatechin (− 2.99 log mol/L) and chlorogenic acid (− 1.198 log mol/L). Based on these findings (per Admetlab), quercetin 3-O-rutinoside is considered the most suitable compound for local pulmonary administration due to its low pulmonary dissolution and permeability.

Considering the idea that a portion of an inhaled drug is deposited in the mouth and eventually swallowed and the fact that some asthma drugs are administered orally, it is therefore important to analyze the oral pharmacokinetics of these compounds. The swallowed portions of these compounds will have pharmacokinetic profiles similar to oral drugs.

### Absorption

The efficient absorption of oral drugs depends on different contributing factors. Lipophilicity is one of the descriptors of absorption. As opposed to the desired exposure in the lungs, an oral drug must have a sufficient level of lipophilicity to aid its movement across the intestine. The (−)-epicatechin, ombuine, and quercetagetin have an optimal level of lipophilicity with log *P* values of 1.142 log mol/L, 3.194 log mol/L, and 1.588 log mol/L. Chlorogenic acid and quercetin 3-O-rutinoside have log *P* values slightly less than the optimal value (0 − 3 log mol/L) and will have a relatively slower movement across the intestinal membrane [61–65]. The lipophilicity and intestinal absorption of the compounds follow the order ombuine > quercetagetin > (−)-epicatechin > chlorogenic acid > quercetin 3-O-rutinoside (Table 1). It is noteworthy that the log *P* value has an impact not only on membrane permeability but also on hydrophobic binding to macromolecules including target proteins, enzymes, and transport proteins [51].

### Distribution

Volume distribution (VD) gives an insight into the actual amount of a drug in systemic circulation after absorption. The VD values of all the compounds are within the optimal range. Comparatively, quercetin 3-O-rutinoside showed the highest VD value (0.754 L/kg), while chlorogenic acid returned the least value (0.351 L/kg) (Table 2). Notably, these two compounds showed a positive plasma protein binding (PPB) with values of 83.81% and 76.17%, respectively, while the other compounds returned values slightly higher than the optimal range (90%). Compounds with values higher than 90% may have a low therapeutic index. Interestingly, all the compounds would not permeate the blood-brain barrier.

### Metabolism

Bioactive active compounds in systematic circulation are transported to the liver where they undergo one or more reactions to increase their activity and make them easily excretable. Cytochrome P450 enzymes are predominantly involved in these reactions. (−)-Epicatechin, chlorogenic acid, and quercetin 3-O-rutinoside are non-inhibitors of the analyzed CYP isoforms. Ombuine is predicted to be an inhibitor of 1A2, 2C9, 2D6, and 3A4 isoforms, while quercetagetin would likely inhibit 1A2 and 3A4 isoforms (Table 3). Cytochrome P450 enzymes are phase 1 reaction orchestrators, in cases where they are inhibited, toxic concentrations of drugs and other xenobiotics accumulate due to inefficient processing and clearance [66].

### Excretion

The clearance and half-life are essential pharmacokinetic descriptors that define the excretion of drugs and they serve as important parameters in establishing drug dosage [51]. (−)-Epicatechin has a high clearance rate with a value of 17.911 ml/min/kg, and quercetagetin returned a moderate clearance value (7.705 ml/min/kg). However, chlorogenic acid, ombuine, and quercetin 3-O-rutinoside showed low clearance (3.251 ml/min/kg, 4.929 ml/min/kg, and 1.349 ml/min/kg, respectively) (Table 4).

### Toxicity

According to the Pro-tox II server, the toxicity predicted revealed that (−)-epicatechin is the least toxic with an LD50 value of 10,000 mg/kg, while quercetagetin is the most toxic of all the top-scoring compounds. Chlorogenic, ombuine, and quercetin 3-O-rutinoside also showed a low toxicity profile (Table 5). Furthermore, none of the compounds is predicted to have a hepatotoxic activity, and they are all shown to be non-toxicants of the respiratory system. This increases their suitability for local pulmonary administration. However, quercetagetin was predicted to have a predicted carcinogenic activity. Nevertheless, none of the compounds showed respiratory toxicity.

### Drug-likeness

The drug-likeness and the bioavailability score of a compound predict the likelihood of a compound being an oral drug. All but quercetin 3-O-rutinoside obeyed the Lipinski rule of five and the GSK rule-based filter. Notably, all compounds obeyed the Pfizer rule. (−)-Epicatechin, ombuine, and quercetagetin had a bioavailability score of 0.55. However, chlorogenic acid had a score of 0.11, and quercetin 3-O-rutinoside had a score of 0.17 (Table 5).

## Conclusions

(−)-Epicatechin, chlorogenic acid, ombuine, quercetagetin, and quercetin 3-O-rutinoside were identified to have high binding affinity and inhibitory potential on chymase, spleen tyrosine kinase, and prostaglandin 2D receptor CRTH2. Although the identified compounds generally showed impressive binding to all the targets, quercetin 3-O-rutinoside is considered the most suitable drug candidate. Further, ADMET studies predicted that these compounds can be employed as oral drugs and could be aerosolized for local pulmonary administration. The current findings suggest the reported compounds could be explored as triple-action inhibitors of the protein targets in the management of asthma. However, further corroborative analyses are recommended.

## Data Availability

All data generated or analyzed during the current study are included in this published article

## Abbreviations

ADMET: Absorption, distribution, metabolism, excretion, and toxicity
AHR: Airway hyperresponsiveness
CPA3: Carboxypeptidase A3
cPLA2: Cytosolic phospholipase A2
CRTH2: Chemoattractant receptor-homologous molecule expressed on Th2 cells
HLF: Human lung fibroblast
IgE: Immunoglobulin E
IL: Interleukins
ILC2s: Innate lymphoid cells
PGD2: Prostaglandin D2
PKC: Protein kinase C
SYK: Spleen tyrosine kinase
